# Clinical diagnosis and management of *Demodex* blepharitis: the *Demodex* Expert Panel on Treatment and Eyelid Health (DEPTH)

**DOI:** 10.1038/s41433-023-02500-4

**Published:** 2023-03-24

**Authors:** Brandon D. Ayres, Eric Donnenfeld, Marjan Farid, Ian Benjamin Gaddie, Preeya K. Gupta, Edward Holland, Paul M. Karpecki, Richard Lindstrom, Kelly K. Nichols, Stephen C. Pflugfelder, Christopher E. Starr, Elizabeth Yeu

**Affiliations:** 1 Wills Eye Hospital, Philadelphia, PA USA; 2 Ophthalmic Consultants of Long Island, Long Island, NY USA; 3 Gavin Herbert Eye Institute, UC-Irvine, Irvine, CA USA; 4 Gaddie Eye Centers, Louisville, KY USA; 5 Triangle Eye Consultants, Raleigh, NC USA; 6 Tulane University, New Orleans, LA USA; 7 University of Cincinnati, Cincinnati, OH USA; 8 Kentucky Eye Institute, Lexington, KY USA; 9 University of Minnesota, Minneapolis, MN USA; 10 School of Optometry, University of Alabama at Birmingham, Birmingham, AL USA; 11 Baylor College of Medicine, Houston, TX USA; 12 Weill Cornell Medicine, New York, NY USA; 13 Virginia Eye Consultants, Norfolk, VA USA

**Keywords:** Eyelid diseases, Eye manifestations

## Abstract

**Background:**

Twelve ocular surface disease experts convened to achieve consensus about *Demodex* blepharitis (DB) using a modified Delphi panel process.

**Methods:**

Online surveys were administered using scaled, open-ended, true/false, and multiple-choice questions. Consensus for questions using a 1 to 9 Likert scale was predefined as median scores of 7–9 and 1–3. For other question types, consensus was achieved when 8 of 12 panellists agreed. Questions were randomized, and results of each survey informed the following survey.

**Results:**

Twelve practitioners comprised the ***D****emodex* **E**xpert **P**anel on **T**reatment and Eyelid **H**ealth (DEPTH). Following 3 surveys, experts agreed that DB is chronic (*n* = 11) and recurrent (*n* = 12) and is often misdiagnosed. Consensus was achieved regarding inflammation driving symptoms (median = 7; range 7–9), collarettes as the most common sign (*n* = 10) and pathognomonic for DB (median = 9; range 8–9), and itching as the most common symptom (*n* = 12). Panellists agreed that DB may be diagnosed based on collarettes, mites, and/or patient symptoms (*n* = 10) and felt that patients unresponsive to typical therapies should be evaluated for DB (*n* = 12). Consensus about the most effective currently available OTC treatment was *not* reached.

**Conclusions:**

The Delphi methodology proved effective in establishing consensus about DB, including signs, symptoms, and diagnosis. Consensus was *not* reached about the best treatment or how to grade severity. With increased awareness, eyecare practitioners can offer DB patients better clinical outcomes. A follow-up Delphi panel is planned to obtain further consensus surrounding DB treatment.

## Introduction

Blepharitis is chronic ocular inflammation primarily involving the eyelid margin, found in approximately 47% of patients presenting for eye examinations [1–3]. Blepharitis frequently disrupts the ocular surface, leading to conjunctivitis, conjunctival erythema, functional tear deficiency, and keratitis. It may also exacerbate symptoms of coexisting ocular surface diseases, including allergic conjunctivitis and aqueous tear deficiency. The chronic nature of blepharitis, its uncertain aetiology, and frequent coexistence of other ocular surface diseases contribute to the challenge of managing affected patients [1]. With no FDA-approved treatments, management of blepharitis includes warm compresses, eyelid hygiene, topical and oral antibiotics, and topical anti-inflammatory agents [1]. Current management strategies may address the symptoms and contributors to blepharitis but not its root cause.

**Fig. 1:**
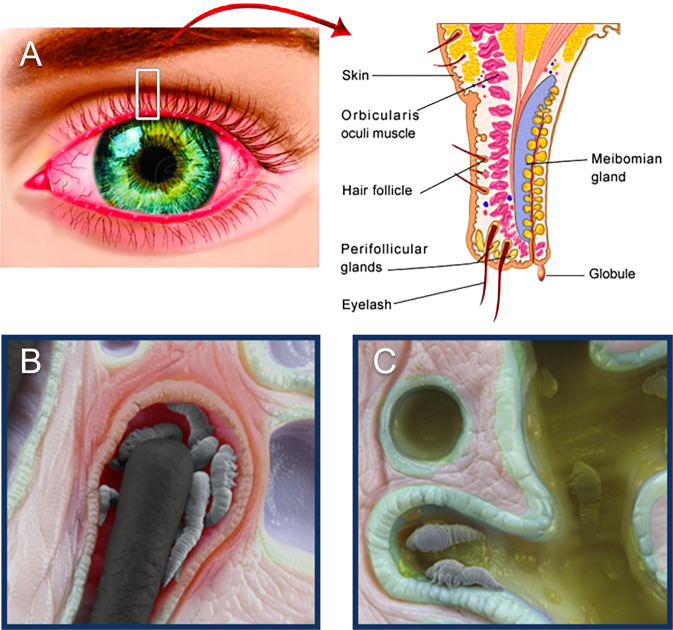
Eyelash schematic and microscopic views of *Demodex* mites. **A** Overview schematic of eyelash and gland structures. **B** *Demodex folliculorum* mites located within the lash follicle. **C** *Demodex brevis* mites located within the meibomian gland. *Images provided courtesy of Tarsus Pharmaceuticals, Inc*.

*Demodex*, a microscopic ectoparasite, is often implicated in blepharitis [4, 5]. Of more than 1600 species of mites collectively known as *Demodex*, two—*Demodex folliculorum* and *Demodex brevis*—inhabit the human body [6]. *D. folliculorum* live in the lash follicle, whereas *Demodex brevis* burrow into the sebaceous and meibomian glands [7]. (Fig. 1) Although the presence of *D. folliculorum* in the eyelashes was first described by Coston over 50 years ago [6], the diagnosis and treatment of *Demodex* blepharitis (DB) remain somewhat challenging, particularly with no FDA-approved treatments currently available.

Both *D.* *folliculorum* and brevis are implicated in DB [8–10], which may represent up to 70% of all cases of blepharitis [5, 11–14]. Considering that a large subset of blepharitis patients are infested with *Demodex*, understanding its role can have significant impact on blepharitis management. Recent research suggests that DB is commonly misdiagnosed or underdiagnosed [15] due to overlap in symptoms with other ocular surface diseases. Thus, there is lack of consensus surrounding the diagnosis, treatment, pathophysiology, and signs and symptoms of *Demodex* blepharitis.

One way to gain consensus is through the Delphi panel methodology [16]. The Delphi methodology, first used by the RAND Corporation, allows experts to achieve consensus utilizing sequential surveys [16]. This approach includes defining a problem, developing questions, selecting experts, administering questionnaires to panellists, performing qualitative and quantitative analyses of responses, and repeating subsequent surveys until consensus is established [17, 18]. This methodology has been used across eyecare with expert panels convened to achieve consensus about ocular allergy [19], cataract surgery [20, 21], thyroid eye disease [22], macular degeneration [23], keratoconus [24], glaucoma [25, 26], dry eye [27, 28], uveitis [29], neurotrophic keratopathy [30], and inherited retinal diseases [31, 32]. To the authors’ knowledge, this is the first Delphi panel to address *Demodex* blepharitis.

## Materials and methods

**Fig. 2:**
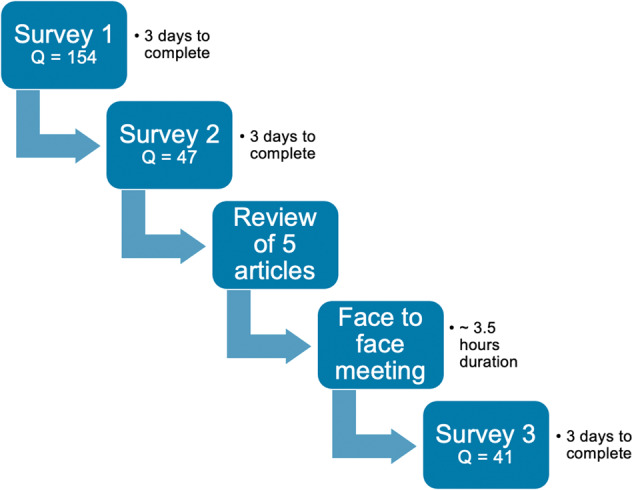
*Demodex* Expert Panel on Treatment and Eyelid Health flowchart. Tasks undertaken as part of the DEPTH process.

A neutral third-party medical communications company was engaged to develop, design, and oversee this Delphi panel (i2Vision, San Diego, CA). Figure 2 shows the steps undertaken. The panel included ophthalmologists and optometrists of both genders from the USA in different types and duration of clinical practice. All individuals were identified as experts in external disease, blepharitis, and ocular surface diseases. A literature search of *Demodex* blepharitis was conducted using key words ‘collarette,’ ‘cylindrical dandruff,’ ‘*Demodex*,’ ‘demodicosis + eye,’ and ‘blepharitis’ for clinical papers published between 2015 and 2021. The search yielded 95 papers related to *Demodex* and *Demodex* blepharitis that were used in survey creation.

**Fig. 3:**
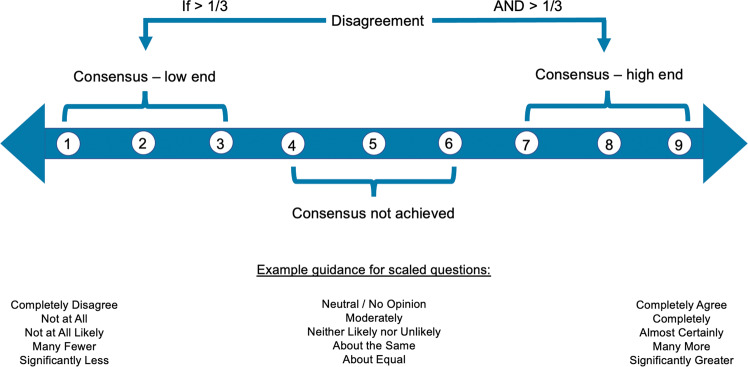
How consensus was defined. A 1–9 Likert scale was selected, and consensus was predefined. Median scores of 1–3 and 7–9 indicated consensus on the low and high ends of the scale, respectively. Median scores of 4–6 indicated no consensus. If more than 1/3 of the panel members selected 1–3 and more than 1/3 of the panel members selected 7–9, this was considered disagreement. For the open-ended questions, including yes/no and numeric answers, consensus was achieved when 8 of 12 panellist answers agreed.

To produce quantifiable results, a 1–9 Likert scale was chosen, and a portion of Survey 1 questions were structured using it. In order to prevent bias, consensus was defined prior to administering the first survey. Median scores of 7–9 and 1–3 indicated consensus at the high and low ends of the scale, respectively. A median score of 4–6 indicated consensus was not achieved, and if >one-third of the panel members selected 1–3 and >one-third of the panel members selected 7–9, this was considered disagreement. For closed-ended questions (including yes/no, true/false, and numeric), consensus was achieved when 8 of 12 panellists agreed (Fig. 3).

Three rounds of surveys were submitted electronically. To minimize bias from “survey fatigue,” questions were randomized for each participant. All questions had to be answered before moving to the next section. The surveys contained intentionally repeated questions worded differently to provide information about consistency of panellists’ responses. For example, 3 questions were asked about the predominant symptom in DB patients but were worded in different ways.

Survey 1, consisting of 154 questions, covered a range of topics related to *Demodex* blepharitis. The survey comprised a combination of scaled, open-ended, and closed-ended questions (yes/no or numeric) with the option for respondents to elaborate via free text. The goal of Survey 1 was to gain general understanding across a range of topics related to DB and which areas consensus existed among panellists. The results of Survey 1 were compiled and analyzed, specifically noting questions and topical areas that achieved consensus.

Survey 2 focused on confirming consensus obtained in Survey 1, both to ensure consistency of responses over time and to gain consensus on additional topics. The 47 closed-ended questions were multiple choice, yes/no, or true/false. Questions were developed based in part on answers provided by the panellists on the Survey 1 open-ended questions. As with Survey 1, the results were compiled and analyzed for consensus.

Following Survey 2, 5 articles about *Demodex* blepharitis were sent to panellists as pre-reading material [5, 11, 14, 33, 34]. Articles chosen were recently published in well-known, geographically diverse journals with audiences of both ophthalmologists and optometrists. Studies in these articles had large patient populations followed longitudinally and focused on DB. A live video meeting was then held to discuss aspects of *Demodex* blepharitis. While the original Delphi methodology relied strictly on surveys to maintain anonymity, the modified Delphi process includes one or more in-person meeting and is more common recently, particularly in healthcare [35–38]. The 3-hour face-to-face meeting aimed to foster discussion among the experts, considering the first 2 surveys and the pre-reading materials. The meeting was moderated, and general themes from Surveys 1 and 2 were presented. The goal was *not* to achieve further consensus, but rather to discuss DB and engage in robust peer-to-peer discourse [39].

After the face-to-face meeting, a third and final survey with 41 closed-ended questions was administered. Questions were based on outstanding areas without consensus from Surveys 1 and 2 and discussion at the live meeting. Following compilation and analysis of Survey 3 results, data from all surveys were combined; the results are presented here.

## Results

### Demographic information of the expert panellists

The response rate for each survey was 100%. Nine ophthalmologists and 3 optometrists formed the ***D****emodex* **E**xpert **P**anel on **T**reatment and Eyelid **H**ealth (DEPTH). Panellists’ mean age was 53.7 years (SD 10.5 years, range 40–73 years), and the average number of years in practice was 23.9 (SD 10.7, range 10–43 years). Four panellists were women (33.3%) and 8 were men (66.7%). Half of the participants practiced in an academic or academic referral setting and the other half in a range of private practice settings. All panellists were from the USA (50% Southeast (*n* = 6), 25% Northeast (*n* = 3), 8% West (*n* = 1), 8% Midwest (*n* = 1), 8% Southwest (*n* = 1)).

In these results, numbers in parentheses indicate how many of the 12 panellists agreed to a statement for closed-ended questions. For scaled questions, because the distribution of responses was generally skewed, the range is reported along with the median. Open-ended questions did not necessarily have a numeric score, but we report patterns that emerged.

### Typical patient population

In general, the panellists deemed DB to be chronic (*n* = 11) and recurrent (*n* = 12). While panellists see *Demodex* in all age groups, they reported seeing it most frequently in people ≥60 years (*n* = 11). An open-ended question yielded 33% of responses indicating no racial predilection, 17% unsure, 42% Caucasian, and 8% African American. When asked what proportion of their *Demodex* patients are male, the responses ranged from 30 to 70%. The proportion of the total female (range 0–80%) or male (range 3–80%) population that has DB also varied widely. There was no link between patients’ socioeconomic status and the presence of DB (*n* = 8). Furthermore, clinicians unanimously agreed that examination for DB should be part of every routine eye exam (*n* = 12).

### Physiology and pathophysiology

The DEPTH panellists unanimously agreed that inflammation is a key result of DB (*n* = 12), with *Demodex* mites and their byproducts triggering the inflammatory cascade (median score 7; range 7–9). The itching accompanying DB, panellists believed, is propagated through non-histamine itch pathways (median score = 7; range 4–9). There was consensus that disease progression starts with increased collarettes leading to inflammation and lid erythema, followed by conjunctival injection, then lid margin thickening/notching/oedema, followed by lash loss/irregularity and potentially corneal staining (*n* = 8). With respect to whether *Demodex* mites are part of the “normal” ocular flora, the panellists achieved consensus surrounding the idea that *Demodex* is a common parasite found on the skin, and when overgrowth occurs it can cause blepharitis (*n* = 11). They also agreed that DB is a chronic recurrent condition in which mites can be eradicated but reinfestation is possible (*n* = 11).

### Signs and symptoms

According to DEPTH, collarettes (cylindrical dandruff) are the most common sign (*n* = 10) and pathognomonic for DB (median score = 9; range 8–9). Panellists agreed that conjunctival injection is common (median score = 7; range 3–8), and lash loss occurs only in patients with severe disease (median score = 7; range 1–9). There was consensus that tear break-up time (TBUT) is impacted by DB (*n* = 8) and that itching is the most common symptom (*n* = 12). The location of the itching and how patients describe it (e.g., eyelid itching vs. just eye itching) can be helpful in implicating *Demodex* as the diagnosis. Panellists felt that the coexistence of dry eye disease (DED) and DB increases the likelihood of itching compared to those with *Demodex* alone (median score = 8; range 5–9). They also reported that patients tend to experience their worst discomfort in the mornings (n = 10). Additionally, the experts felt that the mite count correlates with density/severity of collarettes (median score = 9; range 4–9) and severity of symptoms (median score = 8; range 6–9). Lastly, the DEPTH panel concurred that patients can have *Demodex* infestation and collarettes with or without other clinical symptoms (redness, FBS, itching, epiphora). However, when symptoms are present, other clinical signs are usually present (lid margin redness, madarosis, misdirection of lashes, lid margin oedema).

### Diagnosis and grading

Panellists agreed that slit lamp examination is the most common method used for diagnosing DB (*n* = 12) and that visualization of mites is not necessary to make the diagnosis (median score = 2; range 1–8; note that this question was one which indicated consensus on the scale’s low end). The diagnosis of DB may be based on one or a combination of presence of collarettes, mites, or patient symptoms including itching (*n* = 10). Of the 12 DEPTH experts, 10 indicated that the biggest advance in diagnosis of DB has been understanding that collarettes are pathognomonic for the condition. Initially there was consensus that the terms ‘collarette’ and ‘cylindrical dandruff’ are the same and used interchangeably, however in the third survey, panellists agreed that preferred nomenclature for this clinical sign moving forward, in order to better educate the eyecare community about DB, is collarettes.

Panellists felt that epilation is not necessary (median score = 9; range 5–9), nor is it necessary to count individual mites (*n* = 11). Grading the severity of DB is important and clinically useful (*n* = 11), panellists concurred, but no consensus about a specific scale was reached. To monitor treatment efficacy, the DEPTH panel agreed that tracking the severity or number of lashes with collarettes is more important than the degree of ocular irritation (*n* = 8). The experts indicated that patient education using the words mites, bugs, or microorganisms is helpful (*n* = 12).

### Associated conditions

According to the panel, rosacea has a strong association with DB (*n* = 11) as well as being a risk factor (n = 10). Rosacea was also the most-cited systemic condition seen with *Demodex* blepharitis (*n* = 9). The group agreed that patients with DB may have secondary ocular infections (median score = 7.5; range 2–9) that, when present, are usually bacterial (*n* = 9). Meibomian gland dysfunction (MGD) is often found along with DB, with panellist estimates ranging from 50–100% of patients experiencing both (median = 80%). The DEPTH panel concurred that dry eye occurs in more than half of DB patients (*n* = 11). These experts unanimously agreed that with the overlap in symptoms among *Demodex* blepharitis, dry eye, and MGD, DB may be underdiagnosed or misdiagnosed (*n* = 12). There was also unanimous consent that in addition to treatment-naïve patients, those who have not responded to typical lid disease management should be evaluated for *Demodex* (*n* = 12). While in agreement that no association exists between DB and gastro-intestinal comorbidities (*n* = 10), the panel did not reach consensus about whether systemic conditions like rheumatoid arthritis, other autoimmune conditions, or diabetes may predispose patients to DB.

### External factors

When queried about external factors potentially related to DB, the DEPTH experts agreed that contact lens intolerance correlates with *Demodex* infestation (median score = 7; range 7–9), and lenses should be discontinued until DB is treated, provided the patient is having issues (n = 9). The group also concurred that DB patients should be treated before undergoing ocular surgery (*n* = 11).

### Psychosocial factors

The DEPTH panel felt that DB affects patients’ overall quality of life (median score = 7, range 6–8), and that patients may experience unhappiness or anxiety (median score = 7; range 6–9), as well as insecurity about their appearance (median score = 8; range 6–9). There was consensus that patients find their symptoms more bothersome than the physical appearance or psychological aspect of having “bugs” on their eyelids (*n* = 9).

### Treatment

Collarettes with symptomatic blepharitis, the panel agreed, are indicative of DB and should be treated. Restoring balance to the ocular ecology is the key to managing *Demodex* infestation (median score = 8; range 5–9), and mechanical intervention (e.g., lid scrubs, blepharoexfoliation) is an important part of treatment (*n* = 12). However, consensus about the most effective currently available over-the-counter treatment, in light of lack of any FDA-approved therapies, was not reached. Of the management options for *Demodex* blepharitis available at the time of this panel, the group was about evenly split between blepharoexfoliation and tea tree oil as their primary strategy.

Heat, whether warm compresses, steam-based devices, or radiant heat devices, was deemed to be minimally, marginally, or not useful (*n* = 10). The primary concern of panellists when treating *Demodex* patients is treatment efficacy (*n* = 11) above tolerability or safety. These experts unanimously agreed that blepharitis may be best treated via a decision tree that accounts for clinical signs and patient symptoms (*n* = 12). The panel also agreed that patients with no/minimal symptoms but a moderate number of collarettes would warrant a treatment trial for DB.

Areas of consensus on scaled questions are summarized in Table 1.

**Table 1:** Key areas of consensus on scaled questions.

Area of consensus	Median score	Range
Collarettes are pathognomonic for *Demodex* blepharitis	9	8–9
Epilation is not necessary	9	5–9
Number of mites correlates with density and severity of collarettes	9	4–9
*Demodex* blepharitis may cause insecurity about appearance	8	6–9
Number of mites correlates with symptom severity	8	6–9
Restoring balance to the ocular ecology is the key to managing *Demodex* infestation	8	5–9
More itching is seen in dry eye disease with *Demodex* blepharitis vs. *Demodex* blepharitis alone	8	5–9
*Demodex* blepharitis patients may have secondary ocular infections	7.5	2–9
Contact lens intolerance correlates with *Demodex* infestation	7	7–9
*Demodex* mites and their byproducts such as chitin and digestive enzymes trigger the inflammatory cascade	7	7–9
Inflammation drives symptoms in *Demodex* blepharitis	7	7–9
Itching is caused by non-histamine pathways	7	4–9
Lash loss only occurs with severe *Demodex* blepharitis	7	1–9
^a^Mite visualization NOT necessary to diagnose	2	1–8

Presented here are a selection of the scaled questions administered in Survey 1 for which consensus was achieved. Items are listed from highest degree of consensus. The higher the median score AND the tighter the range, the greater the degree of consensus.

^a^Recall, median scores between 7 and 9 and between 1 and 3 indicated consensus achieved at the high and low ends of the scale, respectively.

## Discussion

Blepharitis is a recurrent condition, often refractory to existing management strategies, and considered a lifelong condition. The current therapeutic endpoint is not to cure but to control or manage the disease. Although reported prevalence of DB varies greatly [5, 11, 12], it is widespread, perhaps even more so knowing that significant numbers are misdiagnosed [15]. The current study is a first step in raising awareness, as the DEPTH panel attempts to establish consensus across areas including hallmark signs and symptoms, diagnosis, and treatment.

The Delphi methodology has proven effective in achieving consensus. While the original process used written surveys, online methods as used here have shown similar validity [33, 40]. Consistent with previous reports of Delphi panels, 3 rounds of surveys were adequate to reach consensus about most of the DB categories surveyed [16, 41]. Khodyakov et al. showed that by including high quality face-to-face feedback and allowing respondents to reconsider their answers after discussion, greater understanding may be achieved [39]. Accordingly, Survey 3 was designed after the live meeting and utilized some discussion topics to help reach consensus in areas previously lacking. A specific example would be questions about the most common symptom in patients with DB. Although consensus was not obtained following Survey 1, following Survey 3 the panel agreed that itching was most common.

For topics like signs and symptoms, associated conditions, and psychosocial effects of DB, consensus was largely achieved in the first 2 rounds. However, for other topics, like prevalence and the primary goal of treatment, consensus was not reached even after the third survey. This is likely not because of the survey itself, but may be more indicative of the lack of high quality epidemiological studies or the differences in panellists’ practice settings and patient populations.

One area of consensus obtained in early rounds was the presence of collarettes indicating *Demodex* blepharitis. These collarettes are gelatinous in appearance and create a cuff around the base of the lash, not to be confused with collarettes formed from *Staphylococcus* which are golden-yellow and scaly in appearance and located more distally on the lash [42]. Coston and others have reported that collarettes are pathognomonic for *Demodex* blepharitis [5, 6, 43]. In both the first and second surveys, the DEPTH panel agreed that collarettes are pathognomonic for DB. The consensus was that panellists would treat patients if collarettes are present, even in the absence of symptoms.

Another sign for which DEPTH panellists obtained consensus was TBUT being impacted by DB. Recent studies by Sędzikowska and Nowomiejska demonstrated that TBUT was significantly shorter in *Demodex* positive patients [44, 45], further emphasizing the impact of DB on the ocular surface.

The panel concurred that when *Demodex* are present, collarettes are present as well. This aligns with Gao et al. who found *Demodex* mites present in all patients with collarettes [46]. Historically, definitive diagnosis of *Demodex* blepharitis relied on the epilation of lashes to visualize and count individual mites. However, due to the impracticality of light microscopy and slide preparation capabilities in an outpatient clinic, along with patient discomfort and increased exam time, the DEPTH panel agreed that since collarettes are pathognomonic for *Demodex* mites, clinicians may confidently move away from the necessity of epilation.

Itching, redness, and tearing are common symptoms reported in patients with DB [25, 47]. Sędzikowska and colleagues reported that 64% of 1499 patients had ocular symptoms, with itching most common (28%), followed by redness (21%) and watering (15%) [46]. The qualitative results in our study mirror these findings. Although itching was not conclusively reported as the primary symptom in the first 2 surveys, at the end of Survey 3, itching emerged as the most common symptom. The panellists also concluded that when patients report itching, specifically eyelid itching, DB should be at the top of the differential list and warrants further evaluation for collarettes.

At the time of this Delphi panel, options reported in the literature for managing DB include tea tree oil, topical or oral ivermectin, and blepharoexfoliation. A recent meta-analysis of 6 clinical studies reported that the effectiveness of tea tree oil for managing DB is unclear, dependent on factors such as oil concentration, dosage, lid hygiene, and compliance [48]. Tea tree oil has reported side effects of ocular irritation, allergy, and contact dermatitis [48–50], and an in vitro study demonstrated it was toxic to human meibomian gland epithelial cells in culture after 15 min of exposure to 1% terpinen-4-ol (T4O). At 90 min, nearly all of the cells died. Even when the concentration was decreased to 0.001%, there was still a marked decrease in cell survival [51].

DEPTH panellists were divided about current preferred *Demodex* management, half using tea tree oil and half preferring blepharoexfoliation, but the group concurred that more research is necessary and new FDA-approved treatment options that target and kill all mites are needed. Panellists agreed that presence of DB negatively impacts patients’ sense of well-being and, potentially, quality of life. The Delphi panel came to consensus that reducing or eradicating mites completely is important. Literature review and the results of our study confirm the need for a more effective, safe, and well-tolerated therapy for *Demodex* blepharitis.

Several conditions such as rosacea, MGD, and DED often occur with DB [52–55]. Since clinically these conditions are often very similar, the panel concurred that DB is frequently underdiagnosed or misdiagnosed. The consensus, therefore, was that all patients presenting for an eye exam should be evaluated for collarettes, especially those with lid abnormalities or those not responding to treatment for DED or MGD. DEPTH panellists shared that slit lamp examination with the patient looking down is simple and easy to incorporate into routine exams.

As with most studies, there are some limitations to the data presented here. The expert panel was composed of 12 members, a relatively small “sample size” that may not accurately reflect diversity among clinicians, practice settings, and patient populations. Even though DEPTH consisted of panellists with known expertise in, passion for, and commitment to advancing knowledge in ocular surface disease, results from this particular group may not be repeatable with different experts. Additionally, there is always potential for bias to be introduced via the survey process, the surveys themselves, the background reading, and even the face-to-face meeting. To mitigate this possibility, steps such as predefining consensus and question randomization were taken to minimize possible sources of bias.

This study is the first to show that the Delphi methodology is effective in establishing consensus surrounding various aspects of DB. Overall, consensus was obtained across numerous aspects of the disease including signs, symptoms, diagnosis, and associated ocular conditions. Consensus was *not* reached on other factors, such as the best therapeutic option and the best way to grade DB. Increased awareness of *Demodex* blepharitis in the eyecare community will raise the level of care received by patients with blepharitis and offer some a more targeted treatment strategy and better clinical outcomes.

Because questions remain regarding the treatment of DB, another Delphi panel is being planned, focusing on treatment.

## Summary

### What was known before

- Blepharitis is chronic eyelid margin inflammation found in approximately 47% of patients presenting for eye examinations.
- Up to 70% of blepharitis cases may be due to *Demodex*infestation.
- Because *Demodex*blepharitis shares many signs and symptoms with other ocular surface conditions, it is often mis- or underdiagnosed.

### What this study adds

- Through a systematic process of literature review, successive surveys, and peer-to-peer discussion, this expert panel came to consensus about many aspects of *Demodex* blepharitis.
- Consensus was reached about the typical; patient, key signs and symptoms, effective examination strategies to best recognize *Demodex*blepharitis, and associated ocular and systemic conditions.
- While there was agreement about some aspects of treatment, further study is warranted to reach consensus on the most effective management strategies.

## Data Availability

The authors declare that the data that supports the findings of this study are available within the article.
